# CD45RC Isoform Expression Identifies Functionally Distinct T Cell Subsets Differentially Distributed between Healthy Individuals and AAV Patients

**DOI:** 10.1371/journal.pone.0005287

**Published:** 2009-04-21

**Authors:** Laurence Ordonez, Isabelle Bernard, Fatima-Ezzahra L'Faqihi-Olive, Jan Willem Cohen Tervaert, Jan Damoiseaux, Abdelhadi Saoudi

**Affiliations:** 1 Institut National de la Santé et de la Recherche Médicale (INSERM) U563, Institut Fédératif de Recherche (IFR) 30, Hôpital Purpan and Université Paul Sabatier, Toulouse, France; 2 Department of Internal Medicine, Division Clinical and Experimental Immunology, Maastricht University Medical Centre, Maastricht, The Netherlands; Karolinska Institutet, Sweden

## Abstract

In animal models of anti-neutrophil cytoplasmic antibody (ANCA)-associated vasculitis (AAV), the proportion of CD45RC T cell subsets is important for disease susceptibility. Their human counterparts are, however, functionally ill defined. In this report, we studied their distribution in healthy controls (HC), AAV patients and in Systemic lupus erythematous (SLE) patients as disease controls. We showed that CD45RC expression level on human CD4 and CD8 T cells identifies subsets that are highly variable among individuals. Interestingly, AAV patients exhibit an increased proportion of CD45RC^low^ CD4 T cells as compared to HC and SLE patients. This increase is stable over time and independent of AAV subtype, ANCA specificity, disease duration, or number of relapses. We also analyzed the cytokine profile of purified CD4 and CD8 CD45RC T cell subsets from HC, after stimulation with anti-CD3 and anti-CD28 mAbs. The CD45RC subsets exhibit different cytokine profiles. Type-1 cytokines (IL-2, IFN-γ and TNF-α) were produced by all CD45RC T cell subsets, while the production of IL-17, type-2 (IL-4, IL-5) and regulatory (IL-10) cytokines was restricted to the CD45RC^low^ subset. In conclusion, we have shown that CD45RC expression divides human T cells in functionally distinct subsets that are imbalanced in AAV. Since this imbalance is stable over time and independent of several disease parameters, we hypothesize that this is a pre-existing immune abnormality involved in the etiology of AAV.

## Introduction

Anti-neutrophil cytoplasmic antibody (ANCA)-associated vasculitis (AAV) constitutes a group of disorders characterized by autoimmune inflammation affecting small- to medium-sized vessels, which leads to vessel occlusion and systemic organ damage [1]. AAV consists of four different disease entities: Wegener's granulomatosis (WG), microscopic polyangiitis (MPA), Churg-Strauss syndrome (CSS), and renal-limited vasculitis. ANCA in these vasculitides are directed against either proteinase 3 (PR3) or myeloperoxidase (MPO). Although the etiology of AAV is not well understood [2], several studies have implicated T cells in the pathogenesis, in particular in WG [3], [4]. More recently, various T cell subsets were found to be either enlarged or functionally impaired, including regulatory T cells (Treg), naive and memory T-cells, Th1, Th17 and Th2 cells [5]–[14].

CD45 is a high molecular weight transmembrane protein with intrinsic tyrosine phosphatase activity. This heavily glycosylated protein is expressed at high level on nucleated cells of the haematopoietic system and is essential for efficient T and B cell antigen receptor signal transduction [15]. Several CD45 isoforms can be generated by alternative splicing of exons 4(A), 5(B) and 6(C) leading to change in the extracellular domain of the molecule [16]. Importantly, polymorphisms and mutations that affect CD45 alternative splicing, and thus isoform expression, have been associated with several human autoimmune diseases [17]–[20]. However, although CD45 alternative splicing is highly regulated and conserved among vertebrates, the function of the different CD45 isoforms is not clear. In the rat, the level of CD45RC isoform expression divides CD4 and CD8 T lymphocytes in two subpopulations. The CD45RC^high^ T cell subset produces preferentially type-1 cytokines, while type-2 and immunoregulatory cytokine production is restricted to the CD45RC^low^ subset [21]–[24]. The relative proportion of CD45RC^high^ and CD45RC^low^ T cell subsets varies between rat strains that differ in their susceptibility to develop immune mediated diseases [22], [23], [25]. Brown Norway (BN) rats, that are prone to develop MPO-ANCA associated vasculitis [26]–[29], have a preponderance of the CD45RC^low^ T cell subset [25]. Importantly, this difference in the proportion of CD45RC^high^ and CD45RC^low^ T cell subsets is genetically controlled by the same chromosomal regions that have been shown to influence the susceptibility to immune mediated disorders [22], [23], [25], [30]. Based on these experimental findings, suggesting that the imbalance between CD45RC^high^ and CD45RC^low^ T cell populations contributes to the susceptibility to vasculitis, we examined the distribution and function of the CD45RC subsets in healthy individuals and AAV patients.

In the present study, we show that CD45RC subsets within the CD4 and CD8 T cell compartments exhibit different cytokine profiles, and that their relative proportion is variable from one individual to another. Interestingly, the proportion of CD45RC^low^ CD4 T cells is strongly increased in AAV patients as compared to healthy controls and patients with systemic lupus erythematosus (SLE). Since this increase is not associated with disease subtype, disease duration or number of relapses, we hypothesize that the observed imbalance between CD45RC^high^ and CD45RC^low^ T cell subsets is a pre-existing phenomenon that may be involved in the etiology of AAV.

## Materials and Methods

### Study population

For analysis of the distribution of CD45RC T cell subsets in peripheral blood, patients were recruited via the outpatient clinic of the Maastricht University Medical Centre (Maastricht, The Netherlands). All AAV patients (n = 38; 21 men and 17 women; median age 57 [range 32–75]) fulfilled the disease definitions as proposed by the Chapel Hill Consensus Conference [31]. Only patients with inactive disease, as evaluated by the Birmingham Vasculitis Activity Score [32], were included. Characteristics of AAV patients are presented in table 1. The SLE patients (n = 20; 4 men and 16 women; median age 40 years [range 22–64 years]) fulfilled the revised criteria of the American College of Rheumatology [33] and had inactive disease at the time of sampling. Patient's spouses were recruited as control subjects (n = 39; 17 men and 22 women; median age 55 years [25–70]). Written informed consent was obtained from all subjects. They were informed about the study and were enabled to ask further information. All subjects had sufficient time to consider participation. This study was approved by the Medical Ethics Committee of the University Hospital of Maastricht. For cytokine analysis on CD45RC T cell subsets, PBMC were obtained from buffy coat preparations from anonymous healthy donors, from the Purpan university hospital blood bank (Toulouse, France).

**Table 1:** Clinical characteristics of AAV* patients

	Total AAV (n = 38)	WG (n = 24)	MPA (n = 6)	CSS (n = 4)	RLV (n = 4)	SLE (n = 20)
**Gender (M/F)**	21/17	12/12	5/1	2/2	2/2	16/4
**Age, median (range) yrs**	57 (32–75)	55 (32–75)	61 (56–75)	54 (45–60)	65 (58–75)	40 (22–64)
**ANCA (PR3/MPO/none)**	24/9/5	20/2/2	3/3/0	0/1/3	1/3/0	-
**Disease duration, Median (range) yrs**	2.6 (0.6–16.3)	3.5 (0.9–16.3)	1.0 (0.6–4.0)	4.2 (0.9–9.4)	0.8 (0.6–1.7)	6 (0.7–28.1)
**Renal involvement (+/**−**)**	18/20	11/13	2/4	1/3	4/0	9/11
**Relapses, median (range)**	1.3 (0–5)	1.9 (0–5)	0 (0–0)	0.8 (0–2)	0 (0–0)	-

* Abbreviations: AAV, ANCA-associated vasculitis; ANCA, anti-neutrophil cytoplasmic antibody; CSS, Churg-Strauss syndrome; MPA, microscopic polyangiitis; MPO, myeloperoxidase; PR3, proteinase 3; RLV, renal-limited vasculitis; WG, Wegener's granulomatosis.

### Antibodies

FITC-, PE-, PE-Cyan5, PE-Cyan7,Alexa 700, Pacific Blue, APC or biotin-conjugated anti-CD4 (RPA-T4), anti-CD8 (RPA-T8), anti-TCRαβ (BW242/412), anti-HLA-DR (LN3), anti-CD25 (4E3), anti-CD28 (CD28.2), anti-CD69 (FN50), anti-CD45RA (HI100), anti-CD45RB (MT4), anti-CD45RC (MT2), anti-CD45R0 (UCHL1), anti-CCR7 (3D12), anti-Foxp3 (PCH101), anti-IL-4 (4D9), anti-IL-10 (JES3-9D7) and anti-IFN-γ (25723.11) mAbs as well APC or PC7-streptavidin and biotinylated MARG-2a were purchased from BD Biosciences (San José, CA), R&D Systems (Minneapolis, MN), IQ Product (Groningen, The Netherlands), Miltenyi (Bergisch Gladbach, Germany), Beckman Coulter (Fulletron, CA) or eBioscience (San Diego, CA).

### Flow cytometry analysis

For immunofluorescence staining, 10^6^ cells were incubated with mAbs for 20 min at 4°C. After washing with phosphate buffered saline (PBS) containing 5% fetal calf serum (FCS) the biotin-labeled cells were incubated with streptavidin-coupled PC7 for 20 min at 4°C, washed twice with PBS/5% FCS. Foxp3 intracellular expression was detected using APC anti-human Foxp3 Staining Set from eBioscience, according to their standard protocol. Data were collected either on a FACS-Calibur (BD Biosciences) cytometer using the CELLQuest™ software (BD Biosciences) for analysis or on a LSR-II (BD Biosciences) cytometer using the DIVA software (BD Biosciences) for analysis.

### T cell subsets purification

PBMCs were prepared by gradient centrifugation (MLS-Ficoll, Eurobio, Les Ulis, France) of buffy coat. Monocytes were removed by plastic adherence and CD4 and CD8 T cells were purified by negative selection using CD4 or CD8 negative isolation kit (Dynal; Oslo, Norway). The percentage of residual CD8 or CD4 T cells after depletion was always less than 0.5 % and the remaining population consisted of 95–98% CD4 or CD8 T cells and 2–5% CD4 CD8 double negative non T cells. The isolation of CD45RC^high^ and CD45RC^low^ CD4 T cell subsets was performed as follows: CD4 T cells were stained with limiting amounts of FITC-conjugated anti-CD45RC mAb and separated into CD45RC^high^ and CD45RC^low^ cells by positive selection after addition of anti-FITC MACS microbeads (Miltenyi). The resulting purity was always more than 92% for CD45RC^high^ and CD45RC^low^ CD4 T cells. For cell sorting experiments, purified CD4 T cells were stained with anti-CD4, anti-CD45RC and anti-TCR mAbs and separated on a Coulter cell sorter (Epics Altra; Beckman-Coulter, Fullerton, CA). The purity of sorted CD45RC^high^ or CD45RC^low^ CD4 T cell subsets was more than 99 %. To purify CD45RA^high^ and CD45RA^low^ CD4 T cell subsets, the CD45RC^low^ CD4 subpopulation, purified by magnetic beads, were labeled with anti-CD45RA mAb and separated on a Coulter cell sorter according to CD45RA expression. The purity of sorted CD45RA^high^ or CD45RA^low^ CD4 T cell subsets was more than 99 %. Similar procedures were used for purification of CD4 CD45RB subpopulations within the CD45RC^low^ subset. CD8 CD45RC T cell subsets were purified by cell sorting after labeling purified CD8 T cells with anti-CD8, anti-CD45RC and anti-TCR mAbs. The purity of sorted CD45RC^high^ , CD45RC^int^ or CD45RC^low^ CD8 T cell subsets was more than 97 % .

### T cell stimulation and analysis of T cell proliferation and cytokine production

The culture medium was RPMI 1640 (Gibco Life Technologies Ltd, Cergy Pontoise, France) containing 5 % of human SAB (Biowest, France), 1% sodium pyruvate, 1% non essential amino acids, 1% L-glutamine, 1% penicillin-streptomycin and 2×10^−5^ M 2-mercaptoethanol. Highly purified CD45RC CD4 or CD8 T cell subsets (10^5^ T cells / well) were polyclonally stimulated in 96 well plates (Falcon, Becton Dickinson) using bound anti-CD3ε (TR66; kindly provided by Dr Valitutti, Toulouse, France) and soluble anti-CD28 mAbs (CD28.2, BD Biosciences). Proliferation was measured by ^3^H-thymidine uptake during the last 18 h of a 24, 48, 72 or 96 h culture period. At various times throughout the culture, supernatants were analyzed for cytokine production using CBA kit (BD Biosciences) or ELISA for IL-17 (eBiosciences). Cytokine production was also assessed by intracellular staining as described [34]. Briefly, CD4 or CD8 T cell subsets were stimulated for 72 h with anti-CD3ε plus anti-CD28 mAbs, then activated with phorbol 12-myristate 13-acetate (PMA) (20 ng/ml, Sigma) plus ionomycin (0.8 µg/ml, Sigma,) in the presence of Monensin (2 µM, Sigma) for 4 h. Cells were harvested, fixed with 2% paraformaldehyde (Fluka Chemie AG, Buchs, Switzerland) and permeabilized with 0.5% saponin (Fluka). Cells were incubated with FITC-labeled antibody to IFN-γ (Beckman Coulter), PE-labeled antibody to IL-4 or IL-10 (BD Biosciences washed, and analyzed by flow cytometry on FACSCalibur or LSRII.

### Statistical analysis

Data are presented as box plot. The Wilcoxon matched-pairs test was used for intra-individual comparison. Linear regression analysis was performed to assess associations between the age and the proportion of CD45RC T cell subsets and to assess the association between the relapse and the proportion of CD45RC T cells subsets. The non-parametric Mann–Whitney U-test was used to compare data from AAV patients, SLE patients and HCs. *, p<0.05; **, p<0.02; p<0.002.

## Results

### The proportion of CD45RC CD4 and CD8 T cell subsets is highly variable in the human population

**Figure 1:**
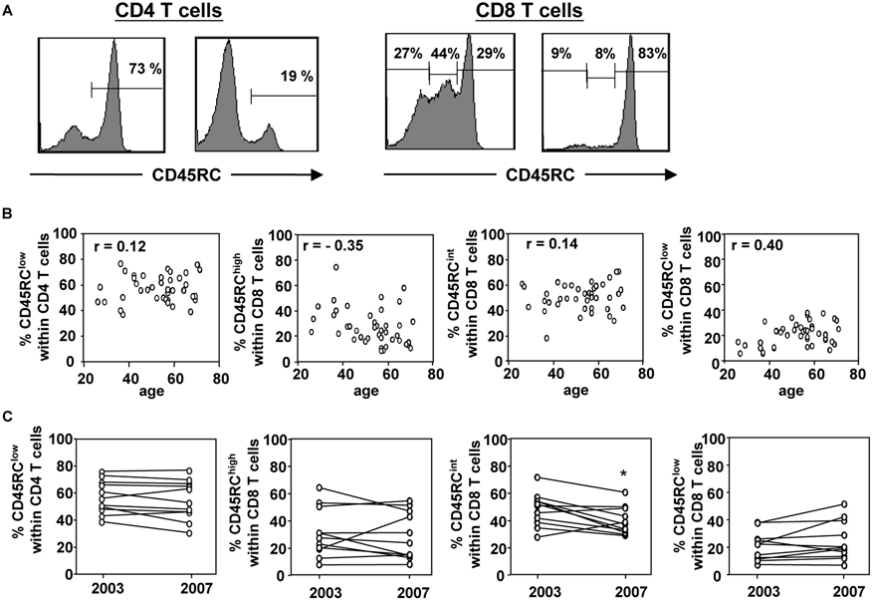
CD4 and CD8 CD45RC T cell subsets distribution in healthy individuals. Peripheral blood leukocytes from 39 healthy individuals (median age 55, range 25–70) were stained with mAbs against CD3, CD4, CD8, CD45RC. (A) The histograms represent the CD45RC expression on CD4 T cells (left panel) and CD8 T cells (right panel) from two healthy individuals showing the inter-individual variability in CD45RC expression. (B) The proportion of CD45RC^low^ CD4 T cells (left panel) and the proportion of CD45RC^high^-CD45RC^int^-CD45RC^low^ CD8 T cells (right panels) are presented according to age of the donors. Each dot represents a separate individual. The r- and p-values were calculated using linear regression. (C) Represent the percentage of CD45RC^low^ CD4 T cells (left panel) or the proportion of CD8 CD45RC T cell subsets (right panel) of 11 individuals at 4 years interval. The p-values were calculated using the Wilcoxon matched-pairs test; *, p<0.05.

The analysis of CD45RC expression on human peripheral blood CD4 T cells by flow cytometry revealed an heterogeneous expression, allowing the definition of two subsets: CD45RC^high^ and CD45RC^low^ (Fig. 1A, left panel). In contrast, the CD45RC expression level on human CD8 T cells revealed a more complex pattern with usually three subsets: CD45RC^high^, CD45RC^int^ and CD45RC^low^ (Fig. 1A, right panel). We analyzed the relative proportion of CD4 and CD8 CD45RC subsets in 39 healthy individuals (22 women and 17 men, median age 56, range 26–71). As shown in Figure 1, this proportion was very heterogeneous for CD4 (median and range for CD45RC^low^: 57% and 37–77%) and for CD8 (median and range for CD45RC^low^: 22% and 7–39%; CD45RC^int^: 51% and 17–71%; CD45RC^high^: 25% and 9–77%). These differences in the relative proportion of CD45RC subsets within the CD4 (Fig. 1B, left panel) and CD8 (Fig. 1B, right panels) T cells were not explained by differences in age. To assess intra-individual variation over time, the proportion of CD45RC T cell subsets were reanalyzed after a period of 4 years in 11 individuals. No significant changes in the proportion of CD45RC T cell subsets were observed during this period except for the CD8 CD45RC^int^ subset (Fig. 1C). Finally, we showed that the observed heterogeneity in the CD45RC subsets was not the result of different numbers of activated T cells, since we found no correlation between the proportion of CD45RC T cell subsets and the percentage of HLA-DR+ cells for CD4 T cells and CD8 T cells (data not shown). Also, the absolute numbers of T cells and the CD4/CD8 T cell ratio were not correlated with the proportion of CD45RC T cell subsets (data not shown). Altogether, these data demonstrate that CD45RC expression identifies different subsets of CD4 and CD8 T cells that are differentially distributed between healthy individuals independently of age or size and activation state of the T cell compartment.

**Figure 2:**
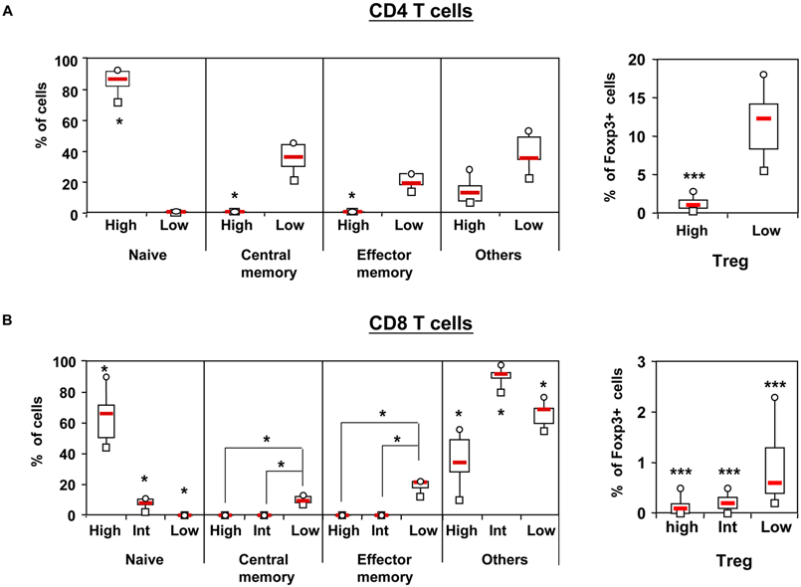
Phenotypic characterization of CD45RC T cell subsets. Peripheral blood leukocytes from healthy individuals were stained with mAbs against TCR, CD4 or CD8, CD45RC, CD45RA, CD45RO and CCR7 (n = 6) or TCR, CD45RC, CD4 or CD8 and Foxp3 (n = 27). Gates were set on CD4 T cells (upper panels) or CD8 T cells (lower panels). Box plot diagrams represent the proportion of naive (CD45RA+CD45RO−CCR7+), central memory (CD45RA−CD45RO+CCR7+), effector memory (CD45RA−CD45RO+CCR7−) and natural regulatory T cells (Foxp3+) within the CD45RC subsets. The group “others” contains both CD45RA+CD45RO−CCR7− and CD45RA+CD45RO+ subsets, subpopulations with ill defined functions. The p-values were calculated using the Wilcoxon matched-pairs test; *, p<0.05; **, p<0.02; p<0.002.

To identify the relation between the CD45RC phenotype and naive T cells (CD45RA+RO−CCR7+), effector memory T cells (CD45RA−RO+CCR7−), central memory T cells (CD45RA−RO+CCR7+) and natural Treg (Foxp3+), we performed 6-color staining flow cytometry. As shown in Fig. 2, the CD4 and CD8 CD45RC T cell subsets are heterogeneous. The majority of CD4 CD45RC^high^ cells are naive cells (87%; range 72–93) whereas the CD4 CD45RC^low^ subset contains central memory cells (median 36%; range 21–46), effector memory cells (median 19%; range 14–26%) (Fig. 2A). Concerning the CD8 T cell compartment, the CD45RC^high^ and CD45RC^int^ subsets contain the majority of naive cells (High: 66%; range 44–90; Int: 8% range 2–20) whereas the CD45RC^low^ subset contains the majority of memory cells (central memory T cells: 10%; range 7–24, effector memory T cells: 22%; range 17–34) (Fig. 2B). A significant proportion of CD4 and CD8 CD45RC T cell subsets contains two subpopulations with ill defined functions i.e; CD45RA+CD45RO−CCR7− and CD45RA+CD45RO+. In addition, we found that the majority of Foxp3+ CD4 and CD8 T cells are contained in the CD45RC^low^ subset (Fig. 2).

### The proportion of CD45RC CD4 and CD8 T cell subsets is differentially distributed between healthy individuals and AAV patients

**Figure 3:**
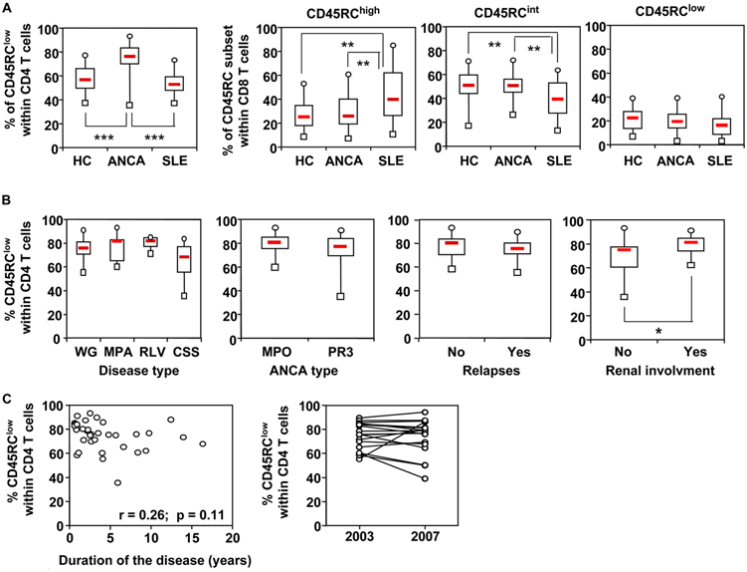
CD45RC T cell subsets distribution in healthy individuals and ANCA patients. Peripheral blood leukocytes from 39 healthy individuals (HC), 38 patients with ANCA-associated vasculitis (AAV), and 20 patients with systemic lupus erythematosus (SLE), were stained with mAbs against CD3, CD4, CD8, CD45RC. (A) The proportion of CD45RC^low^ CD4 T cells (left panel) and the proportion of CD45RC^high^-CD45RC^int^-CD45RC^low^ CD8 T cells (right three panels) are presented as box plot diagrams for each study population. The p-values were calculated using the Wilcoxon matched-pairs test; p<0.05; **, p<0.02; ***, p<0.002. (B) The proportion of CD45RC^low^ CD4 T cells are presented according to disease subtype (WG, Wegener's granulomatosis; MPA, microscopic polyangiitis; CSS, Churg-Strauss Syndrome; RLV, renal limited vasculitis), type of ANCA specificity (MPO, myeloperoxidase; PR3, proteinase 3), renal involvement (no: no kidney disease; yes: kidney disease), and relapses (no: no relapse; yes: relapses). Data are presented as box plot diagrams for each study population. The p-values were calculated using Mann Witney U test; *p<0.05. The proportion of CD45RC^low^ CD4 T cells are presented according to duration of disease (C, left panel). The proportion of CD45RC^low^ CD4 T cells of 18 AAV patients (13 WG, 3 MPA, and 2 RLV patients) at 4 years interval (C, right panel).

The analysis of CD45RC T cell subsets in the peripheral blood of patients with AAV, all in clinical remission, revealed a strong predominance of the CD45RC^low^ subset within the CD4, but not the CD8 T cell compartment (Fig. 3A). Interestingly, we did not observe this increased proportion of CD45RC^low^ CD4 T cells in patients with SLE, another chronic systemic autoimmune disease (Fig. 3A). The percentage of CD45RC CD4 T cells was not different between patients with the distinct disease entities of AAV (WG, MPA, CSS, and renal limited vasculitis), MPO− or PR3-ANCA, or number of relapses (Fig. 3B). Interestingly, the proportion of CD4 CD45RC^low^ subset was significantly higher in AAV patients with renal involvement (Fig. 3B, right panel). Finally, the observed increased proportion of the CD45RC^low^ CD4 T cells in AAV patients was not influenced by the duration of the disease and was stable during 4 year follow-up (Fig. 3C).

### The level of CD45RC expression identifies two subsets within human CD4 T cells with differential cytokine production

**Figure 4:**
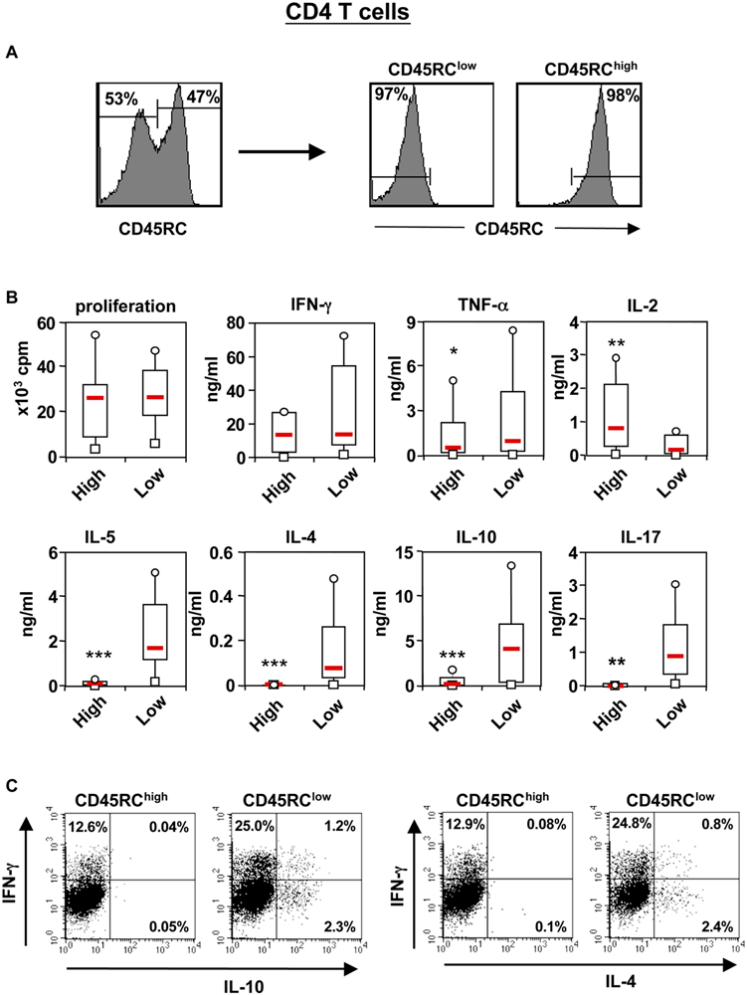
Cytokine profile of human CD45RC CD4 T cell subsets. (A) Representative example of the purification of CD4 CD45RC T cell subsets. Results are shown as histograms for CD45RC expression on CD4 T cells before (left histogram) and after CD45RC subsets purification (right histograms). The values within the histograms represent the percentage of CD45RC T cell subsets. (B) Purified CD45RC^high^ (High) and CD45RC^low^ (Low) CD4 T cell subsets, were stimulated in vitro with plate-bound anti-CD3 and anti-CD28 mAbs. The supernatants were collected at 72 h of culture and analyzed for the presence of cytokines using the CBA kit and Elisa. The results obtained in 20 healthy individuals are presented as box plot diagrams. The p-values were calculated using the Wilcoxon matched-pairs test; *, p<0.05; **, p<0.02; ***, p<0.002. (C) For intracellular measurement of cytokines, purified CD4 CD45RC^high^ and CD45RC^low^ T cells were stimulated and stained using FITC-labeled anti-IFN-γ mAb and PE-labeled anti-IL-4 or anti-IL-10 mAbs. The results are expressed as dot plot representing IFN-γ/IL-4 or IFN-γ/IL-10 production by CD4 T cell subsets. The values within the plots represent the fraction of CD4 T cells producing the indicated cytokine. The results are representative of three independent experiments.

In order to characterize the function of CD45RC^high^ and CD45RC^low^ CD4 T cell subsets, we determined their cytokine profile. For this purpose, we purified these sub-populations from peripheral blood of 20 healthy individuals using magnetic beads. The purity was always higher than 92% (Fig. 4A). Purified CD45RC CD4 T cell subsets were then stimulated *in vitro* in an antigen-presenting cell independent system using plate bound anti-CD3 mAb in the presence of soluble anti-CD28 mAb. Initial experiments showed that the peak of cytokine production was reached after 3 days of stimulation (data not shown). Upon this *in vitro* stimulation, both T cell subpopulations proliferated equally well, but produced different cytokines (Fig. 4B). The type-1 cytokines, IL-2, TNF-α and IFN-γ, were produced by both subsets, but the CD45RC^high^ population produced more IL-2 (Fig. 4B). In contrast, IL-17, IL-10 and the type-2 cytokines (IL-4, IL-5) were mainly produced by the CD45RC^low^ CD4 T cells (Fig. 4B). Similar results were also obtained when CD45RC subsets were highly purified by flow cytometry (>99%), thus excluding a possible contribution of contaminating cells in these differences (data not shown). Intracellular staining confirmed the above results and showed that IFN-γ was produced by both the CD45RC^high^ and CD45RC^low^ subsets, while IL-4 and IL-10 producing cells were mainly contained within the CD45RC^low^ subset (Fig. 4C). In addition, these experiments showed that the majority of IL-4 or IL-10 producing cells did not produce IFN-γ (Fig. 4C). We also analysed CD4 T cells according to the expression of CD45RA isoform. We showed that the majority of CD45RC^high^ subset expresses also high levels of CD45RA isoform. In contrast, the CD45RC^low^ population is heterogeneous and contains both CD45RA^high^ and CD45RA^low^ subsets (Fig. S1A, S1B). After stimulation with anti-CD3 and anti-CD28 mAbs, we showed that purified CD45RC^low^CD45RA^high^ CD4 T cells and CD45RC^low^CD45RA^low^ CD4 T cells exhibited a similar pattern of cytokine production as total CD45RC^low^ CD4 T cell subsets (Fig. S1C and S1D). Similar phenotypic and functional studies were obtained when CD45RB was used instead of CD45RA (data not shown). Since the CD45RA^high^ (and CD45RB^high^) cells within the CD45RC^low^ subset produced IL-4, IL-5 IL-10 and IL-17, we conclude that CD45RC expression is more reliable than CD45RA (and CD45RB) expression to identify human CD4 T cells that are responsible for type-2, IL-17 and regulatory cytokine production. Altogether, these data demonstrate that CD45RC expression identifies two human CD4 T cell subsets with different cytokine profiles. The CD45RC^high^ subset produce mainly type-1 cytokines while T cells responsible for IL-17, IL-10 and type-2 cytokine production are mainly contained within the CD45RC^low^ subset.

### The level of CD45RC expression identifies three subsets within human CD8 T cells with differential cytokine production

**Figure 5:**
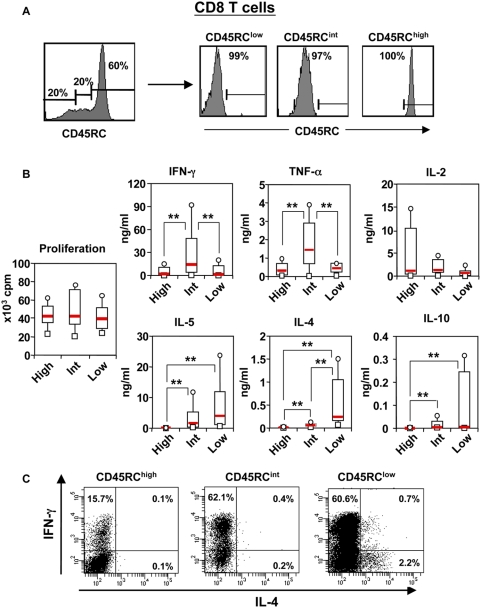
Cytokine profile of human CD45RC CD8 T cell subsets. (A) Representative example of the purification of CD45RC^high^, CD45RC^int^ and CD45RC^low^ CD8 T cell subsets. Results are shown as histograms for CD45RC expression on CD8 T cells before (left histogram) and after CD45RC subsets purification (right histograms). The values within the histograms represent the percentage of CD45RC T cell subsets. (B) These sub-populations were stimulated in vitro with anti-CD3 and anti-CD28 mAbs. The supernatants were collected at 96 h of culture and analyzed for the presence of cytokines using the CBA kit and Elisa. The results obtained in 12 healthy individuals are presented as box plot diagrams. The p-values were calculated using the Wilcoxon matched-pairs test; **, p<0.02. (C) For intracellular measurement of cytokines, purified CD8 CD45RC T cell subsets were stimulated and analyzed for intracytoplasmic cytokines as indicated in the legend of figure 4. The results are representative of three independent experiments.

We also studied the cytokine repertoire of CD45RC^high^, CD45RC^int^ and CD45RC^low^ CD8 T cell subsets. These three sub-populations were purified from peripheral blood of 12 healthy individuals using flow cytometry and stimulated *in vitro* using plate bound anti-CD3 mAb in the presence of soluble anti-CD28 mAb. The purity was always higher than 97% (Fig. 5A). Initial experiments showed that the peak of cytokine production was reached after 4 days of stimulation (data not shown). All three CD45RC CD8 T cell subsets produced the type-1 cytokines, IL-2, TNF-α and IFN-γ, but the CD45RC^int^ population produced more TNF-α and IFN-γ (Fig. 5B). In contrast, IL-4, IL-5, and IL-10 were mainly produced by the CD45RC^low^ and CD45RC^int^ CD8 subsets, with the CD45RC^low^ population producing higher amounts (Fig. 5B). IL-10 was produced only by 4 individuals among 12 tested and IL-17 was undetectable (data not shown). Intracytoplasmic staining confirmed the above results and showed that IFN-γ was produced by all three CD8 T cell subsets, while IL-4 producing cells were mainly contained within the CD45RC^low^ subset (Fig. 5C). Here again, the majority of IL-4 producing cells did not produce IFN-γ (Fig. 5C). Altogether, these data demonstrate that CD45RC expression divides human CD8 T cells into three subsets with differential cytokine production and that the CD8 T cells responsible for type-2 cytokine production and IL-10 production are mainly contained within the CD45RC^low^ and CD45RC^int^ subsets.

## Discussion

In the present study, we show that the level of CD45RC expression on human CD4 and CD8 T cells identifies functionally distinct subsets that differ by their cytokine profile and stage of differentiation. In addition, the proportion of these subsets is diverse within the human population and this diversity is not related with age or the state of T cell activation. Finally, we show that the proportion of CD45RC^low^ CD4 T cells is significantly increased in patients with AAV, but not in SLE, as compared to healthy controls. This increase concerned only the CD4 T cell compartment and appeared independent of AAV subtype, ANCA specificity, number of previous relapses, and duration of disease.

The observation that remission can be induced in AAV patients by drugs specifically targeting T cells strongly suggests a pivotal role of T cells in the pathogenesis of this disorder [3], [35], [36]. In addition, involvement of T cells is suggested by granuloma formation in the lesions and by the presence of isotype-switched autoantibodies, which is compatible with an antigen-driven and T helper cell-dependent autoimmune response. Furthermore, in an animal model of MPO-ANCA associated vasculitis, it was demonstrated that T cells play a pivotal role in the pathophysiology of the disease [37]. Our present study shows that AAV patients in remission harbor an increased proportion of CD45RC^low^ CD4 T cells that is stable over time and independent of disease duration and subtype of AAV. In animal models, T cell activation induces a persistent down-modulation of CD45RC expression, only when the antigen is continuously presented to the immune system [38], [39]. Therefore, the increased proportion of CD45RC^low^ CD4 T cells in AAV patients may be indicative of an ongoing strong antigenic stimulus. In line with this hypothesis, it has been shown that WG patients exhibit a low frequency of naive CD4 T cells [9] and high number of CD4 effector memory cells [13]. Moreover, patients with vasculitis often have increased serum markers of T cell activation [40], [41], and increased percentages of activated T cells [7], both during active disease and in remission. This could be explained by a failure of effectively control T cell activation since a defective suppressive function of circulating Treg has been shown in WG patients [12]. In addition, genetic polymorphisms in genes encoding the inhibitory molecules for T cell activation (CTLA-4, PD1, and PTPN22) have been defined as a risk factor for AAV [42], [43].

It remains to be established whether the increased proportion of CD45RC^low^ CD4 T cells, as observed in AAV patients, is secondary to the disease process, or is a pre-existing phenomenon that contributes to the susceptibility to develop AAV. Since this increase is independent of the treatment (by comparing AAV to SLE patients), the duration of disease or number of previous relapses, we would rather favor the second hypothesis. This is further supported by evidence obtained in animal models. LEW rats, which have a preponderance of CD45RC^high^ T cells, develop preferentially type-1 mediated disorders. In contrast, BN rats, that harbor high amounts of CD45RC^low^ T cells, preferentially develop heavy metal-induced type-2 immune-mediated disorders and MPO-ANCA associated vasculitis [25]–[28]. In addition, the differential distribution of CD45RC subsets between LEW and BN rats is genetically controlled by a locus on chromosome 9 that co-localizes with a 120 kb interval controlling susceptibility of BN rats to develop heavy metal-induced immune-mediated disorders [22], [23], [25], [30] (our unpublished data). Based on these animal model's data, we would like to propose the hypothesis that the imbalance in CD45RC T cell subsets, as observed in AAV patients, may be a risk factor for developing disease.

The next question is how the high frequency of CD45RC^low^ CD4 T cells could influence the development of AAV. It is clear from our study that the CD45RC^low^ CD4 T cell compartment is composed of distinct T cell subsets, including both effector and central memory T cells. Interestingly, a persistent expansion of effector memory CD4 T cells has been described in WG [13], suggesting that the increased proportion of CD45RC^low^ CD4 T cells preferentially affects the effector memory compartment. With respect to the type of effector cells involved, it is of great interest to note that in animal model of type-2 cytokine dependent heavy metal-induced immune disorders, the depletion of CD45RC^high^ T cells exacerbates disease, while adoptive transfer of this subset has a protective effect [44], [45]. This could be due to the differential cytokine production by these T cell subsets. In the current study, we show that IL-17 and type-2 cytokines are exclusively produced by CD45RC^low^ T cells, while type-1 cytokines are produced by both subsets, in agreement with our previous findings in rats [22], [23], [25]. Interestingly, our study also shows that the proportion of CD45RC^low^ CD4 T cell subset is higher in patients with renal involvement, in agreement with the pathogenic potential of this subset. This is in strong concordance with studies showing that Th17 cells are identified in the vasculitic lesions [46] and a skewed distribution of Th2 and Th17 cells in WG patients after either antigen-specific stimulation or polyclonal activation [11], [47], [48]. In addition, IL-17 plays an important role in recruitment and activation of neutrophils, a characteristic feature of AAV disease. How expression of the CD45 isoform could influence cytokine profiles of CD4 T cells is not clear. However, it has been documented that CD45 modulates signalling through diverse receptors affecting cytokine production and response to cytokines [49], [50].

The induction of AAV is multifactorial, with an interplay of environmental factors including silica, bacterial and viral infectious agents, medication, and genetic predisposition, all creating the environment for the development of disease [2]. Our current data suggest that the increased proportion of CD45RC^low^ CD4 T cells may also contribute to the susceptibility to AAV. From animal studies, the relative proportion of CD45RC subsets is genetically controlled by the same genetic interval that also controls several immune-mediated disorders [22], [23], [25], [30]. Although it remains to be determined whether the balance between CD45RC subsets in humans is also genetically controlled, the identification of the gene(s) involved will give new insight in the etiology and pathogenesis of AAV.

## Supporting Information

Figure S1 Differential cytokine production by human CD45RA CD45RClow CD4 T cell subsets (A) CD4 T cells from 18 healthy controls were stained for the expression for CD45RA and CD45RC isoforms. The results are presented as correlation between CD45RAlow and CD45RClow T cells subsets (r = 0.8; p<0.001). (B) Dot plot showing CD45RC and CD45RA expression by CD4 T cells from 2 different donors with different profiles. (C) CD45RChigh and CD45RClow CD4 T cell subsets were purified by flow cytometry. CD45RClow CD4 T cells were stained with anti-CD45RA mAb and separated by flow cytometry into CD45RAhigh and CD45RAlow subsets. (D) These sub-populations as well as total CD45RClow CD4 T cells (white bars) and CD45RChigh CD4 T cells (black bars) were stimulated in vitro with plate-bound anti-CD3 and soluble anti-CD28 mAbs. The supernatants were collected after 72 h of culture and analyzed for the presence of cytokines using the CBA kit. These results are representative of 2 experiments from two different healthy individuals.
(9.73 MB TIF)
