# Supplementary figures and images for: CD45RC Isoform Expression Identifies Functionally Distinct T Cell Subsets Differentially Distributed between Healthy Individuals and AAV Patients
Source: PLoS One. 2009 Apr 21;4(4):e5287. doi: 10.1371/journal.pone.0005287 (PMC2668071; doi:10.1371/journal.pone.0005287)

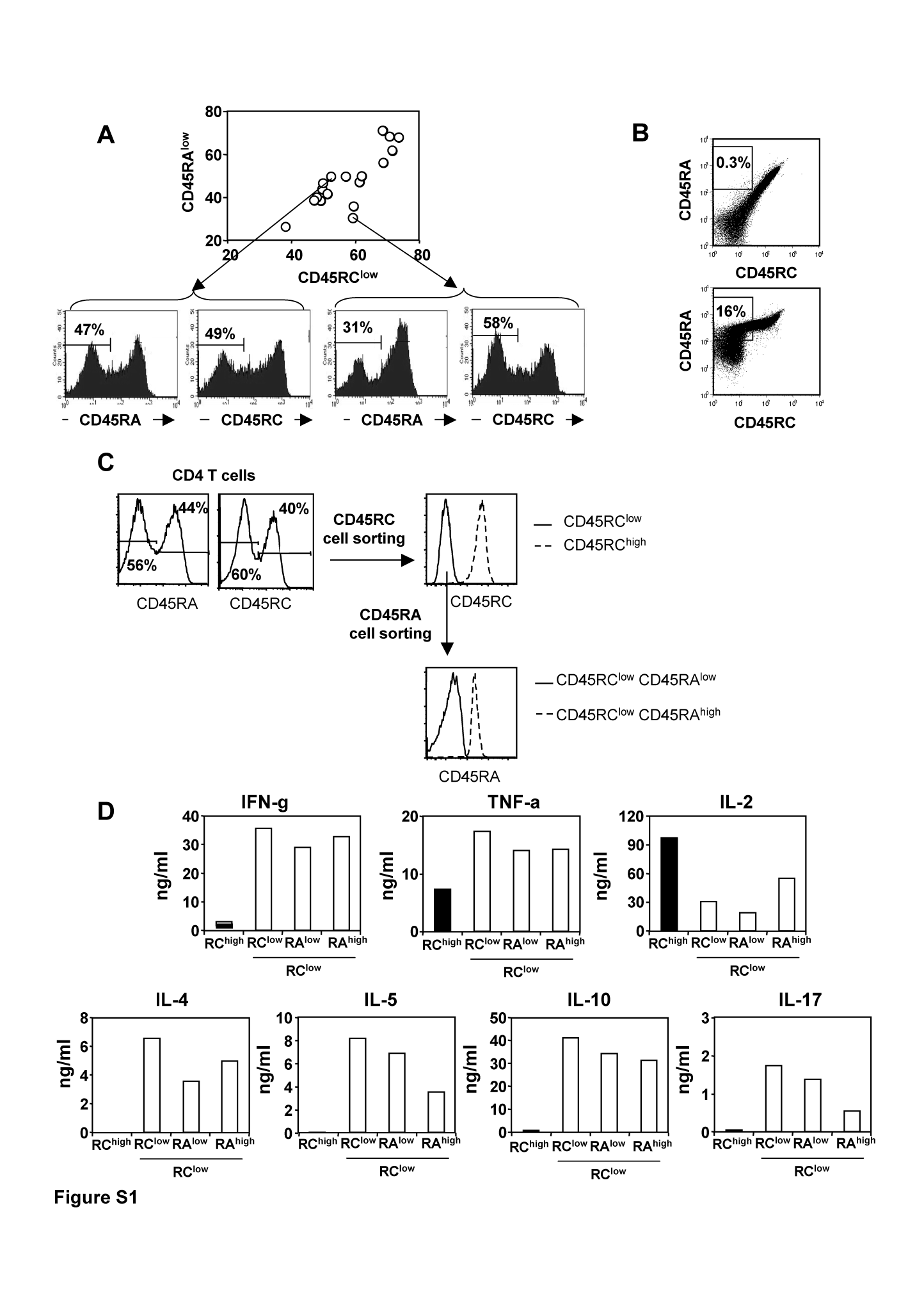

Supplement: Figure S1 — Differential cytokine production by human CD45RA CD45RClow CD4 T cell subsets (A) CD4 T cells from 18 healthy controls were stained for the expression for CD45RA and CD45RC isoforms. The results are presented as correlation between CD45RAlow and CD45RClow T cells subsets (r = 0.8; p<0.001). (B) Dot plot showing CD45RC and CD45RA expression by CD4 T cells from 2 different donors with different profiles. (C) CD45RChigh and CD45RClow CD4 T cell subsets were purified by flow cytometry. CD45RClow CD4 T cells were stained with anti-CD45RA mAb and separated by flow cytometry into CD45RAhigh and CD45RAlow subsets. (D) These sub-populations as well as total CD45RClow CD4 T cells (white bars) and CD45RChigh CD4 T cells (black bars) were stimulated in vitro with plate-bound anti-CD3 and soluble anti-CD28 mAbs. The supernatants were collected after 72 h of culture and analyzed for the presence of cytokines using the CBA kit. These results are representative of 2 experiments from two different healthy individuals. (9.73 MB TIF) [file pone.0005287.s001.tif]
